# (Un)Sustainable transitions towards fast and ultra-fast fashion

**DOI:** 10.1186/s40691-023-00337-9

**Published:** 2023-05-25

**Authors:** Tulin Dzhengiz, Teresa Haukkala, Olli Sahimaa

**Affiliations:** 1 Department of Strategy, Enterprise and Sustainability, Faculty of Business and Law, Manchester Metropolitan University, BS5.32 Business School, All Saints, M15 6BH Manchester, UK; 2 Department of Innovation Management & Entrepreneurship (MIE) i³-CRG — Management Research Centre, Innovation Interdisciplinary Institute (UMR CNRS 9217) ÉCOLE POLYTECHNIQUE Drahi Xnovation Center, Bureau 86.20.51 - 1er étage Avenue Coriolis, 91128 Palaiseau, France; 3 Aalto University School of Business, Ekonominaukio 1, 02150 Espoo / P.O. Box 21210, 00076 Aalto, Finland

**Keywords:** Multi-level perspective, Sustainability transitions, Unsustainability, Institutional logics, Framing contests, Fashion industry

## Abstract

Due to pressing sustainability challenges, the fashion industry is undergoing tremendous change. Surprisingly, even though the unique context of fashion presents an opportunity for scholars to explore the (un)sustainable transitions, this context has yet to receive the attention of transition scholars. Our article explores fashion transitions and develops a conceptual framework demonstrating this transition's multi-level and multi-dimensional interactions. We draw on three literature areas: multi-level perspective (MLP) of sustainable transitions, institutional logics and framing contests. We then introduce a conceptual framework and illustrative examples from the industry and demonstrate the tensions between positive and negative environmental and social sustainability developments at the niche, regime and landscape levels. We show that while many positive developments can be seen in the regime players through the adoption of corporate sustainability initiatives, new business models and collaborations, more attention should also be given to some adverse developments. Overall, we contribute to the literature by exploring fashion transitions, an under-explored context, and by demonstrating the complexity of interactions due to the diffusion of heterogeneous institutional logics and framing contests between players.

## Introduction

The fashion^1^ industry is now considered the second most polluting after oil extraction and production (Diabat et al., 2014, p. 1713). Scholars highlighted the industry’s environmental impacts, such as excessive water use and water pollution (Abbas et al., 2020), GHG emissions from processing fossil fuels (Franco, 2017), and the use of hazardous chemicals (Khurana & Ricchetti, 2016). The industry is responsible for 10% of annual global carbon emissions, equalling international flights and maritime shipping emissions, and is estimated to surge more than 50% by 2030 (World Bank, 2019). Moreover, scholars underlined the industry’s negative societal impacts, such as poor working conditions (Haug & Busch, 2015), health and safety issues (Cesar da Silva et al., 2021), abuses of human rights that include child labour and modern slavery (Peake & Kenner, 2020; Thorisdottir & Johannsdottir, 2020). The awareness of these environmental and social sustainability challenges triggered many changes in the industry towards sustainable and ethical fashion (Alptekinoglu & Orsdemir, 2020; DiVito & Bohnsack, 2017; Goldsworthy et al., 2018; Mishra et al., 2020; Moorhouse & Moorhouse, 2017). Still, the historical development of the industry towards fast fashion and the recent developments towards ultra-fast fashion present negative unsustainable transition trends (Buchel et al., 2018; Maloku, 2020). Thus, it is possible to observe both pro-sustainability (positive) and unsustainable (negative) patterns in the transition journey of the fashion industry. We, therefore, conceptually explore these developments drawing on the sustainability transitions literature and a commonly used framework in that domain: the multi-level perspective (MLP).

MLP is one of the most influential frameworks to explain social transformations that elaborate both the bottom-up and top-down dynamics and the multi-level nature of change (El Bilali, 2019a; Geels, 2002, 2010, 2011, 2020; Hörisch, 2015; Markard & Truffer, 2008; Walrave et al., 2018). Several scholars investigated the conflicting relationships and resistance to change in transitions (Hess, 2016, 2018, 2019; Lee & Hess, 2019), often drawing on the broader literature of social movements and framing (Benford, 1993; Benford & Snow, 2000), institutional theory (Fuenfschilling & Truffer, 2014; Geels, 2020) and institutional logics (Franco-Torres et al., 2020; Runhaar et al., 2020; Smink et al., 2015a, 2015b). Scholars either studied how macro-level institutional structures have debilitated transition pathways; or focused on the micro- and meso-level framing efforts to change the institutional logics (Dobson, 2019; Strambach & Pflitsch, 2018). In recent years, scholars have emphasised the complementarity of institutional logics and framing and combined these lenses to understand and explain institutional inertia and change simultaneously (Ansari et al., 2013; Gray et al., 2015; Purdy et al., 2017).

In this paper, to shed light on the fashion industry's transition, we develop a framework that integrates institutional logics and framing with the interactions in MLP. Herein, drawing on Heinze (2020), we view the fashion system as a complex system that includes material elements (fabrics, factories and retailers), meanings (motivations and emotions) and competencies (capabilities for business, fashion design, and sustainability and responsibility). We specifically focus on the institutional logics and framings associated with the meanings dimension of this fashion system to explain the complexities of multi-level interactions, and we provide various examples to demonstrate the intricacies of fashion transition. Therefore, theoretically, we join the recent scholarly conversation on the current research agenda of transitions (Köhler et al., 2019) by framing contests and logics (Gray et al., 2015; Purdy et al., 2017) into the transitions literature (Geels, 2010; Schot & Kanger, 2018; Welch & Yates, 2018; Zolfagharian et al., 2019). We underline the lack of attention on unsustainable transitions and join others who problematised the sustainability assumption of transitions literature (Susur & Karakaya, 2021). Therefore, we answer the call of Antal et al. (2020), who invited transition scholars to study unsustainable trends, which have received scant attention thus far. Most importantly, we contribute to the literature by conceptually exploring fashion transitions. The fashion context has been largely ignored in transition studies and rarely explored using the lens of framing and institutional logics (Ozdamar Ertekin et al., 2020). By doing so, we start a meaningful conversation on unsustainable transitions in under-studied contexts like fashion.

The remainder of this paper is as follows. In Theoretical Background, we briefly review the literature about MLP in sustainability transitions, institutional logics, and framing. The following section integrates these specific literature areas and builds a conceptual framework while showcasing examples from fashion. In the Discussion, we summarise our findings and compare them with extant research. In Conclusion, we summarise our contributions, offer future research guidance and outline how practitioners can utilise our framework in various ways.

## Literature Review

### (Un) Sustainability in Fashion

The fashion industry “accounts for approximately $2 trillion in global revenue” (Hiller Connell & Kozar, 2017, p. 1). With the dominance of fast fashion, speed-to-market has increased tremendously over the last two decades (Bhardwaj & Fairhurst, 2010), and clothing production almost doubled over the last 15 years (Freudenreich & Schaltegger, 2020). While the industry's economic contribution to our societies is significant, the industry also presents some unique challenges.

In 2015, the fashion industry used 79 billion cubic water; to give further perspective, only a single T-shirt requires 2700 L of water to produce (European Parliament, 2021). Only textile dyeing and finishing are responsible for 20% of global water pollution, and 0.5 million tonnes of microplastics are released every year to the oceans due to washing clothes made of synthetic fibres (European Parliament, 2021). Therefore, many scholars indicated the environmental impacts of this industry as excessive water use, wastewater and water pollution (Abbas et al., 2020; Cesar da Silva et al., 2021; de Oliveira Neto et al., 2019; Jia et al., 2020; Søndergård et al., 2004) and plastic pollution (Goldsworthy et al., 2018; Leal Filho et al., 2019; Moorhouse & Moorhouse, 2017). The industry is also responsible for 10% of global greenhouse gas emissions (European Parliament, 2021), thus the attention on air pollution (Jia et al., 2020; Niinimäki & Hassi, 2011). Due to the nature of fast fashion and increasing overconsumption (Jin & Shin, 2021), clothes are treated as disposable, and fast fashion is also responsible for tremendous waste and waste-related emissions and toxicity (Rossi et al., 2020; Stål & Corvellec, 2018).

Fashion presents a context whereby the consumers play a tremendous role in determining trends (Lee & Ha-Brookshire, 2018; Vehmas et al., 2018). However, due to globalisation and various trade agreements, material sourcing requires global vertically disintegrated value networks (Mellick et al., 2021; Taplin, 2014). It is known that a large portion of labour-intensive clothing production takes place in developing economies (Morris & Barnes, 2009, p. 2). Thus, the end consumer is often far removed from the producers, making it difficult for the consumers to be aware of issues in this global value chain. These extended global value chains make transparency and traceability difficult for the end producer and consumers. Thus, many social sustainability issues in global textile and clothing value chains are unfortunately ignored. These issues include poor working conditions, labour rights, low wages, child labour, and modern slavery (Carrigan et al., 2013; Joergens & Barnes, 2006; Kennedy et al., 2017; Mair et al., 2016; Ozdamar Ertekin et al., 2020; Peake & Kenner, 2020; Pedersen & Gwozdz, 2013; Thorisdottir & Johannsdottir, 2020) and cancer risks due to carcinogenic human toxicity (Haug & Busch, 2015; Søndergård et al., 2004; UNEP, 2020).

Some scholars highlighted sustainability as a megatrend affecting the landscape businesses operate (Park & Kim, 2016). Public attention to microplastics, climate change, and modern slavery has created waves of change in the industry. Depending on their ethical and moral stance, consumers started to demand more environmentally and socially sustainable products, which affected the fashion incumbents’ approach to sustainability challenges (Blasi et al., 2020; Hong & Kang, 2019; Lee et al., 2015). Scholars argue that change in this industry would come “both from end-consumers who prefer sustainable offerings and from businesses (of all sizes) who need to offer products and services that will enable more sustainable consumption” (Turunen & Halme, 2021, p. 2). As a result of increasing consumer demand for more sustainable offerings, incumbents develop interventions in the “design, development, production, distribution, marketing, and consumption of goods may impact multiple stakeholders and simultaneously generate profit for individual companies” (Gaskill-Fox et al., 2014, p. 1). For instance, they develop and source sustainable fibre alternatives such as bamboo (Muthu, 2017; Nayak & Mishra, 2016). Fast fashion incumbents integrate sustainability “in all levels of the business (products, technologies, services, new business models, organization model and relationship with stakeholders)” (Niinimäki, 2015, p. 4). They aim to move towards closed-loop supply chains to cooperate with their suppliers to avoid “damaged products, scraps, and unsold fashion products [going] to a landfill … to properly reuse, remanufacture, and recycle all of them so that some value can be re-generated” (Choi & Li, 2015, p. 15,401). They develop multi-disciplinary teams to design products drawing on specialist knowledge in various areas, including sustainability knowledge (Claxton & Kent, 2020). Still, these efforts are often critiqued and not viewed as sufficient due to greenwashing concerns (Kennedy et al., 2017; Niinimäki, 2015).

Many scholars hope that the true change in fashion will come from niche players and turn to sustainable entrepreneurship (DiVito & Bohnsack, 2017; Heinze, 2020; Kozlowski et al., 2018; Poldner et al., 2016). Here, the expectation is to see novel business models that integrate environmental and social sustainability principles into the core business. Often, such business models move their orientation from profit-making to triple-bottom-line principles (Choi & Li, 2015; Hockerts & Wüstenhagen, 2010), introducing social equity and nature preservation into organizational decision-making (Elkington, 2013).

Developing a multi-level perspective of transitions in this changing and complex industry is beneficial. As our summary of the changes in the fashion above shows, the transition of the industry requires macro (landscape), meso (regime), and micro-level developments (niche). We, therefore, draw on the literature on the multi-level perspective (MLP) in sustainability transitions.

### Multi-level perspective (MLP) in sustainability transitions

Transition studies analyse large-scale sociotechnical changes such as technology and practice changes, policies, and relationships between different societal players and institutions (i.e. values and meaning systems) (Geels, 2002; Rip & Kemp, 1998). There are many different frameworks that transition scholars have developed over time (El Bilali, 2019a; Lachman, 2013); one has been very influential: the Multi-level Perspective (MLP). MLP consists of three analytical concepts: niche, regime, and landscape (Geels, 2002, 2005, 2010; Schot & Geels, 2008). According to Schot and Geels (2008, p. 545), “the core notion of MLP is that transitions come about through interactions between processes at different levels: (a) niche innovations build up internal momentum, (b) changes at the landscape level create pressure on the regime, (c) destabilisation of the regime creates windows of opportunity for niche innovations”.

#### Landscape

The landscape forms the macro-level of the MLP. It sets the scene as “the exogenous environment beyond the direct influence of niche and regime players (e.g. macro-economics, deep cultural patterns, macro-political developments)” (Schot & Geels, 2008, p. 545). The landscape is characterised by relatively slow developments that may take decades of change (El Bilali, 2019a; Raven et al., 2010; Schot & Geels, 2008). A recent review clearly demonstrates that landscape is a “background—a scale with no activities”; thus, “it is difficult to define players at the landscape level since their definition in the MLP does not allow for it” (Fischer & Newig, 2016, p. 6). It is, however, important to emphasise that changes at the landscape level inform the regime and niche players and shape their behaviours. Here, changes at the landscape level mean general technological and cultural trends that are exogenous to the players but shape how they think and behave by providing them with the dominant logics (Fischer & Newig, 2016).

#### Regime

The regime forms the meso-level in the MLP. Regimes refer to the dominant practices, technologies and rules that enable and constrain communities' activities (Geels, 2002). Rip and Kemp (1998, p. 338) defined a technological *regime* “as the grammar or rule set comprised in the complex of scientific knowledge, engineering practices, production process technologies, product characteristics, skills and procedures, and institutions and infrastructures that make up the totality of a technology”. Geels (2002) proposed the term *socio-technical regime,* which underlines the incumbent sociotechnical system that the niche potentially affects or replaces.

It is possible to identify two types of players in the regime. One is the dominant regime players, the incumbents of a particular industry (Fischer & Newig, 2016). The second type is a part of the regime to challenge these incumbents, such as societal pressure groups like NGOs interacting with dominant regime players (Fischer & Newig, 2016).

#### Niches

Niches form the micro-level in the MLP (Geels, 2005; Rip & Kemp, 1998). According to Raven et al., (2010, p. 1), niches “ are a 'space' or 'location' that is protected from the dominant regime, enabling players to develop an innovation, (2) they form the micro-level of technological and social change, (3) they are a new and relatively unstable set of rules and institutions for innovative practices, (4) they are experimental projects, (5) they are a constellation of structures, culture and practices, and (6) they are the variation environment for radical innovations”.

Niche players often replace regime practices and norms (Schot & Geels, 2008). The niche concept is often used positively and is a counterpart to regime problems (Raven et al., 2010). However, niche players may have diverging or conflicting expectations or even no expectations for regime change and, at times, become sites of dispute and consensus (Lazarevic & Valve, 2020; Smith et al., 2010). We problematise the overly optimistic framing of niches and propose a distinction between *negative* and *positive impact niches*.

We define *positive impact niches* as locations where radical pro-social and pro-environmental innovations develop and, given the right incentives, can grow and replace the regime practices. Positive impact niches provide a breathing space for players who make path-breaking innovations and impact the regime to shift towards sustainability. Their innovations are deemed essential and thus need nurturing and protection from the dominating incumbents of the regime. An example of such niches is solar PV technology firms (Smith et al., 2014).

On the contrary, we define *negative impact niches* as locations where radical innovations may develop and replace the regime practices towards unsustainability, leading to a negative environmental impact on the socio-ecological systems. While also aiming at regime shifts, negative impact niches hurt the sustainable transition agenda. Some may welcome such entrants due to their path-breaking innovations. However, they may create unintended consequences on the system if these negative niche players replace the regime (Farla et al., 2012). Similar to our *negative impact niche*, Næss and Vogel (2012) referred to 'unsustainable niches' and emphasised that “the players promoting [unsustainable technologies] and the vested interests they represent seem to be somewhat overlooked in the transition theory literature” (Næss & Vogel, 2012, p. 43). Therefore, Koistinen et al. (2018) underlined the importance of regime players to keep such unsustainable niches outside the gate of regimes, highlighting the multi-level interactions within the MLP. Similarly, Lazarevic and Valve (2020) question which niches should be nurtured and on what grounds.

The MLP framework has received several criticisms to elaborate further on the idea of agency, politics and power (El Bilali, 2019a, 2019b; Svensson & Nikoleris, 2018). Here, one crucial item missing in common MLP frameworks is that niche and regime players are often portrayed as lacking the agency to change the structures that shape them (Fuenfschilling & Truffer, 2014). Here, we point to the literature on institutional logic and framing as helpful ways to integrate further with the MLP framework.

### Diffusion of institutional logics

*Institutional logics* are a “socially constructed, historical pattern of material practices, assumptions, values, beliefs and rules by which individuals produce and reproduce their material subsistence, organise time and space and provide meaning to their social reality” (Thornton & Ocasio, 1999, p. 804). They shape societal structures, field-level practices, organizational forms, and the attentional focus of individuals (Laasch, 2018; Thornton & Ocasio, 1999; Thornton et al., 2012). The distinction between market and sustainability logic is most relevant for conceptualising sustainability transitions.

*Market logic* drives players to maximise shareholder value (Ashraf et al., 2017; De Clercq & Voronov, 2011; Saz-Carranza & Longo, 2012). Dominated by market logic, players aim to maintain their legitimacy by avoiding fines, keeping in line with customer demands, complying with regulatory frameworks, and prioritising profit maximisation through cost reduction and operational improvements (Dahlmann & Grosvold, 2017). Some scholars describe these logics as “transaction-oriented, with profit, self-interest and shareholder value being key for the agency” (Weisenfeld & Hauerwaas, 2018, p. 912).

*Sustainability logic* drives players to prioritise environmental and social value creation and preservation of the natural environment and societal well-being (Corbett et al., 2015; Dahlmann & Grosvold, 2017; Rousseau et al., 2014). Herein, by sustainability, we mean organizations' combined efforts to achieve economic prosperity, environmental quality and social equity simultaneously (Dyllick & Hockerts, 2002). Herein, when we say sustainability logic, we consider both the environmental and social aspects of sustainability. Generally, sustainability logic does not appear the same at for-profit firms as at non-profits. This is due to the institutional complexity created by the *heterogonous exposure to multiple logics* (i.e. market and sustainability logics simultaneously) (Laasch & Pinkse, 2019). Indeed, Haffar and Searcy (2019) demonstrate market-led (business case), value-based and holistic manifestations of sustainability logic in businesses, while Laasch and Pinkse (2019) use the metaphor of 'leopard's spots' to describe institutional complexity.

Institutional logics are exogenous to the players and form the societal antecedents (macro-level) of individual and organizational behaviour (Friedland & Alford, 1991). In the MLP framework, the landscape, as the exogenous environment, would host the complex and multiple institutional logics guiding the regime and niche players' meaning-making systems. On their own, however, institutional logics are “analytically removed from the more active struggles over meaning and resources” (Lounsbury et al., 2003, p. 72), which is why it is essential to explain the framing and framing contests in the MLP framework.

### Framing and framing contests

*Framing* is the act of meaning construction for a specific phenomenon and “involve[s] the ways in which individuals use language or other symbolic gestures in context either to reinforce existing interpretive frames or to call new frames into being” (Cornelissen & Werner, 2014, pp. 18–19). Framing is “effective in challenging dominant logics and legitimating new organizational forms” (Gurses & Ozcan, 2015, p. 1713) and, therefore, a way of driving institutional change (Litrico & David, 2017).

When different players promote different framings, this may lead to *framing contests* (Gurses & Ozcan, 2015; Kaplan, 2008; Pesqueira et al., 2019), which is defined as a “struggle over meaning that attempts to influence the interpretative schemes of players involved in a given situation” (Gurses & Ozcan, 2015, p. 1713). For instance, managers can actively try and shape organizational outcomes through framing contests, often evident when some ideas are vetted, and others are followed due to debates between members that promote different frames (Kaplan, 2008). Similarly, entrepreneurs and incumbents also use framing contests as a mechanism through which they “can battle to enable or disable institutional change” (Gurses & Ozcan, 2015, p. 1704).

Players aim to replace dominant logic through framing since it “involves deliberate communicative acts that shape how individuals perceive, understand, and attach meaning to a given issue” (Bach & Blake, 2015, p. 1). Players convey their perception of reality by using discursive strategies to promote an issue and engage others in taking action for this issue (Benford & Snow, 2000). They give sense to issues to influence others' sensemaking using framing strategies such as symbolic language, metaphors, stock phrases, and idioms (Logemann et al., 2019). For instance, entrepreneurs actively use various framing strategies in their interactions with communities, trade associations and networks to push their solutions forward and convince incumbents and regulators toward their ends. Overall this causes a field-level effect and institutional change, sometimes not intentionally but through their battles of legitimation (Gurses & Ozcan, 2015).

Framing, and more explicitly, framing contests, are interactional and dynamic; they reflect power relations and ongoing negotiations, thus addressing the need for the MLP framework to incorporate agency, politics and power (El Bilali, 2019a). Different players propose different frames, leading to framing contests which is a “politically charged process of meaning construction” (Kaplan, 2008, p. 730).

Six new framings challenge the current fast fashion regime by going beyond eco-efficiency: consistency, degrowth-sufficiency, ethical, slow, circular and sharing, as shown in Table 1. While some pro-sustainability framings overlap, proponents of these different framings sign up for different actions to shift the regime.

**Table 1:** Framing categories for sustainable fashion

Category	Definition
Consistency framing	“Aims to align the materials used in production with material flows that are common in nature” (Freudenreich & Schaltegger, 2020, p. 1)
Degrowth-sufficiency framing	Highlights that “clothing consumers [to] aim to meet their needs rather than fulfil all wants while avoiding (material) consumption as much as possible” (Freudenreich & Schaltegger, 2020, p. 4)
Ethical framing	Underlines the necessity of “fashionable clothes that incorporate fair trade principles with sweatshop-free labour conditions while not harming the environment or workers” (Joergens & Barnes, 2006, p. 361)
Slow framing	“Aims to reduce the number of trends and seasons and encourages quality production in order to increase the value of garments, all in contrast to disposable fashion” (Ozdamar Ertekin & Atik, 2014, p. 57)
Circular and zero-waste fashion	Aims to create closed-loop systems by bringing two strategies: “short-life closed-loop garments and long-life user engagement strategies both have an extending effect on materials in the value-chain, by either keeping products in use over multiple cycles in perpetuity or by extending the single-use cycle of a product over time” (Goldsworthy et al., 2018, pp. 49–50). This framing became more popular in recent years as both within academia and amongst fashion practitioners as the focus shifted to developing recycling, reusing, repurposing business models (Dzhengiz et al. 2023)
Sharing framing	Is about collaborative consumption, which is “ultimately about people sharing and collaborating to meet certain needs” through the creation of “fashion libraries [which] remain a niche activity, driven by enthusiastic entrepreneurs working on a voluntary basis” (Pedersen & Netter, 2015, pp. 258–273)

## The illustrative case of (un)sustainable fashion transitions

**Fig. 1:**
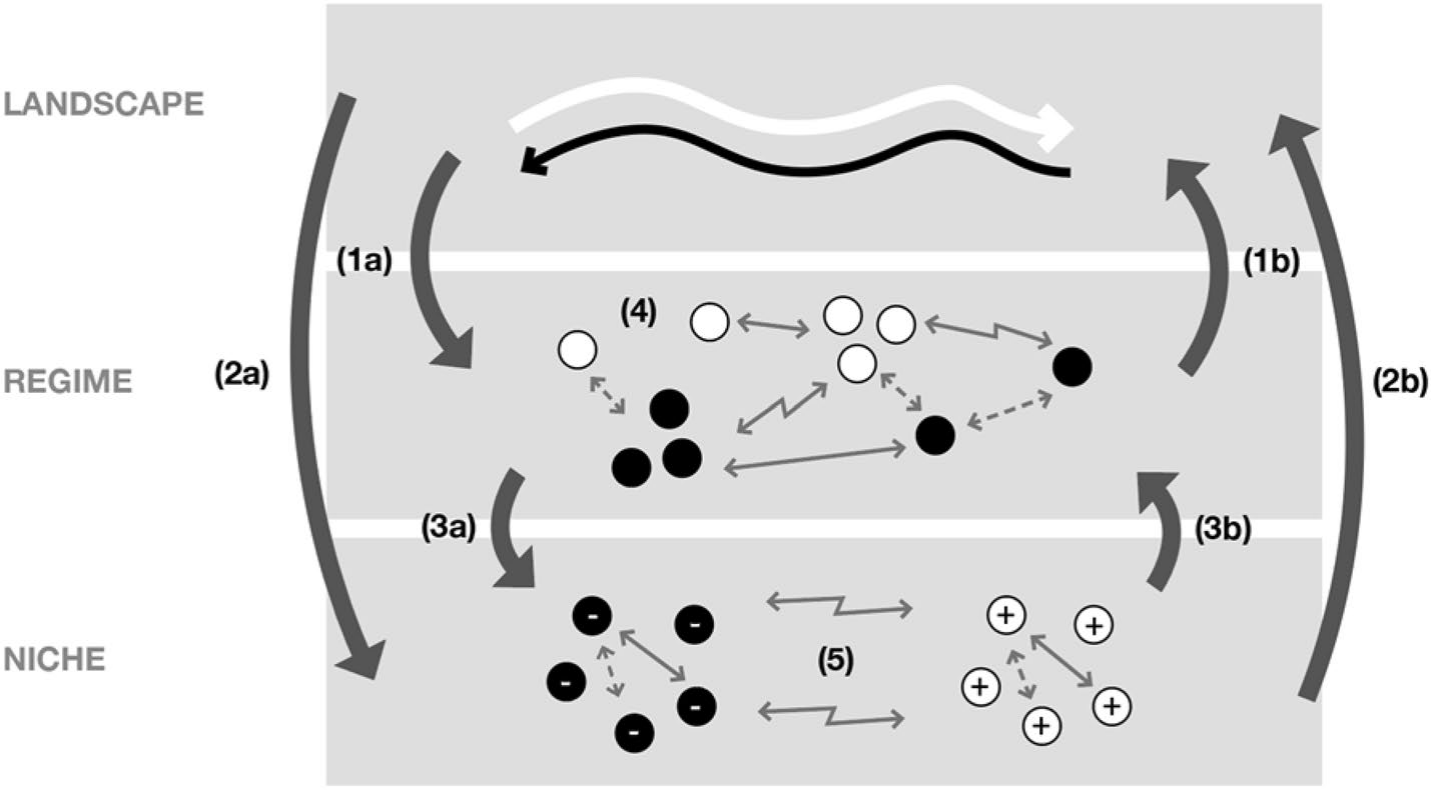
Our conceptual framework **1a** The impact of landscape on regime level. **2a** The impact of landscape on niche level. **3a** The impact of regime on niche level. **1b** The impact of regime on landscape level. **2b** The impact of niche on landscape level. **3b** The impact of niche on regime level. **4** Regime interactions (between different regime actors). **5** Niche interactions (between different niche actors). Framing contest, Cooperative framing, Competitive framing. *White arrow*, Landscape developments for sustainability. *Black arrow*, Landscape developments for unsustainability. +, Positive impact niches. -, Negative impact niches. *Solid arrow* is used to describe cooperative framing, *Dotted arrow* is used to describe competitive framing, *Z shaped arrow* is used to describe the framing contests

This section combines all the concepts introduced in the previous sections in a single encompassing conceptual framework. Drawing on various examples from the fashion context, we conceptualise five interactions in the MLP framework which we further explain through institutional logics and framing contests. As, Fig. 1 shows, these interactions are between the landscape and regime, landscape and niche, regime and niche, between different regime players, and between niche players.

### Landscape-regime interactions

#### Landscape-Regime Interactions (1a)

Currently, landscape change is occurring due to global trends such as demographic developments (population growth and increasing global wealth), the increased use of resources, the consumerist culture, digitalisation, and the urgency to respond to environmental problems and climate change (Buchel et al., 2018, pp. 12–13). We draw on these changes in fashion and explain how these landscape changes have affected the dominant regime and institutional logics.

Before the nineteenth century, it was possible to characterise the clothing industry with general material scarcity and hand-made production, which meant highly-priced pieces of clothing that were expensive to purchase (Ozdamar Ertekin et al., 2020). The Industrial Revolution enabled a significant change. Thanks to the industrialisation wave, which led to the development of the first textile factories in Britain, an industry dominated by tailors and the logic of profession (tailoring) transitioned into the ready-made clothing industry with reduced costs, making clothes easier to access than before (Godley, 2013).

Still, Haute Couture defined the trends for a long time (Ozdamar Ertekin et al., 2020) even though the clothing industry's industrialisation continued. Until the 1980s, the industry was organised to respond to customer demands forecasting their expectations for two seasons, a much slower market speed when compared to today (Bhardwaj & Fairhurst, 2010). Today's giants, Zara and H&M, were considered regional niche players then. In this period, the regime was also a complex field whereby multiple logics such as the logics of the profession (tailoring), art/design and market competed. Amongst others, however, the logic of art/design was central, “mostly concerned with creating innovative and influential trends,” and was associated with “small-scale, labour-intensive production of artistic products” (Ozdamar Ertekin et al., 2020, p. 1451).

Another wave of change in the industry increased market speed tremendously, starting as a niche and quickly gaining the fashion regime's dominance: fast fashion. In the 80 s, fast fashion—“low-cost clothing collections based on current, high-cost luxury fashion trends” (Joy et al., 2015, p. 275)—gained dominance. This meant the “standard turnaround time from the catwalk to consumer of six months (…) [has been] compressed to a matter of mere weeks by such companies as H&M and Zara, with heightened profits to match” (Joy et al., 2015, p. 275). H&M and Zara developed business models aligned with the market logic, increased speed to market, reduced costs and improved price performance (Fuenfschilling & Truffer, 2014; Ozdamar Ertekin et al., 2020). They integrated the design and sales and outsourced production to low-wage suppliers in the developing world (Crofton & Dopico, 2007). The market logic dominated fast fashion model diffused as these models proved a financial success, forcing even the previous Haute Couture players to integrate practices of market logic further to remain legitimate. Gradually, the regional niche players of the 60 s and 70 s—like Zara and H&M—became dominant regime players.

Scholars suggest that “the salience of the influence of market logic, in particular, has risen over the last 30 years” (Thornton et al., 2012, p. 12). “Market logic spread over many socio-technical systems when neoliberal politics became popular in western countries during the 1980s” (Franco-Torres et al., 2020, p. 36). Not surprisingly, this also corresponded with the rise of fast fashion as the regime. As the trade quotas for textiles and clothing were removed in the 90 s, textile value chains changed fundamentally (Mair et al., 2016). Fast fashion utilised these global supply chains that provided cheap labour and access to markets, leading to “the exploitation of Third World labour for the benefit of the leading Western brands” (Simona Segre, 2015, p. 45).

Unfortunately, the fast fashion transition brought societal and environmental problems worth mentioning. The globalisation of the textile value chains came at a high societal price as workers in the developing world suffered poor conditions in unsafe sweatshops with long working hours and low pay (Heinze, 2020; Ozdamar Ertekin et al., 2020; Simona Segre, 2015). Fast fashion has been associated with child labour, modern slavery, and significant health and safety problems (Lueg et al., 2015; Peake & Kenner, 2020), as we listed earlier. Thus, these issues led to the rise of *social sustainability logic in fashion*, initially dominating the niche players and later also adopted by some regime players.

Fast fashion has also created a system whereby disposability became favourable over durability (Joy et al., 2015), creating a throwaway culture resulting in mountains of waste (Freudenreich & Schaltegger, 2020; Niinimäki et al., 2020; Thorisdottir & Johannsdottir, 2020). Overall, the rise of fast fashion led to higher energy and water consumption levels, chemical pollution, soil degradation, and increased carbon footprint (Shirvanimoghaddam et al., 2020), thus leading to the rise of *environmental sustainability logic in fashion*, also initially dominating the niche players and later adopted by the regime players.

The negative impacts of fast fashion came under the spotlight when a tragic field reconfiguring event occurred: the collapse of Rana Plaza, which killed 1129 workers and left 2,500 injured (Lohmeyer & Schüßler, 2017; Williamson & Lutz, 2019). Rana Plaza shook the grounds of the *dominant market logic and reinforced the legitimacy of sustainability logic* further. The workers who lost their lives were producing for well-known brands involved in fast fashion, such as Walmart and Primark (Reinecke & Donaghey, 2015). The Rana Plaza tragedy made it visible that the dominant fashion regime was built on the assumption of infinite growth, strengthened by the craze to buy affordable clothing and the industry's fragmented structure that was based on globalisation and power imbalance between the Global North and South (Buchel et al., 2018).

The Rana Plaza tragedy, therefore, triggered regime changes. For instance, H&M implemented a Conscious Action sustainability initiative to use and promote ecological materials, cleaner production processes, and conduct consumer awareness campaigns (Shen, 2014). H&M's sustainability manager Giorgina Waltier recently argued that “the fashion industry cannot continue to operate in the way it does currently; our planet does not have the resources” (Chan, 2019). However, most incumbents are yet to go beyond eco-efficiency framing, which “aims to either reduce resource use to produce the same output or produce more clothes with a given amount of resources as input” (Freudenreich & Schaltegger, 2020, p. 1).

Post-Rana plaza, policy environment and the proactive regime players needed to enact *sustainability logic to remain legitimate*. Sustainability logic diffused into the regime through certification schemes and norm-setting institutions (Smink et al., 2015a, 2015b). To a greater degree, sustainability logic is also diffused thanks to policy and governance initiatives that affect regime reconfigurations (Roesler & Hassler, 2019). For instance, the Bangladesh Accord and the Sustainability Compact could be governance developments post-Rana Plaza (Bair et al., 2020; Siddiqui et al., 2020). Similarly, Fashion Revolution – founded post-Rana Plaza collapse, started to create further awareness by developing transparency ratings for brands’ supply chains (Monroe, 2021).

Based on the above, we argue the following:

*Interaction 1a—Landscape diffuses market and sustainability logics to the regime players through various norm-setting institutions, providing institutional complexity at the regime level.*

#### Regime–landscape interactions (1b)

Regime players can either engage in framing to legitimise or delegitimise sustainable practices. It is difficult to imagine that a single regime player would impact the landscape, characterised by long-term historical developments and trends. However, coalitions formed between different regime players can influence the regime either positively or negatively, depending on the implications in terms of sustainability impact. In other words, it is possible to differentiate between regime coalitions that are protagonists or antagonists based on their sustainability orientation (Lempiälä et al., 2019). While niche players may also be involved in such coalitions, in the examples from fashion, the coalitions mainly were dominated by the regime players.

Regime incumbents in the fashion system formed a few protagonist coalitions and networks. Fashion for Good, initially founded by the C&A foundation—today called Laudes foundation—engages in social entrepreneurship and philanthropy as part of the European clothing group C&A (Laudes Foundation, 2021). Fashion for Good legitimises circular pro-sustainability framing backed by other industry incumbents like Kering, PVH Corp., and Adidas (Fashion for Good, 2021). Partnership for Sustainable Textiles—founded in October 2014 in response to the deadly accidents in textile factories in Bangladesh and Pakistan, was initiated by the German Federal Minister for Economic Cooperation and Development. This multi-stakeholder platform aims to guide the development of international agreements, setting frameworks and defining principles, allowing members to push their ethical fashion framing (Partnership for Sustainable Textiles, 2021). Global Fashion Agenda promotes circular fashion framing post-Covid crisis (D’Adamo & Lupi, 2021). These protagonist coalitions show that regime players provide their (often aligned with eco-efficiency, as explained earlier) framing to others in their field, legitimise themselves and their practices, and gradually aim to shift dominant logic in alignment with their interests, affecting landscape-level in the long run.

The regime, however, is a complex field. Hence, while fast-fashion incumbents join the networks and platforms mentioned above in alignment with the environmental and social sustainability logic, such initiatives are often critiqued for buried corporate interests and greenwashing (Partzsch et al., 2019). Some scholars argued that such coalitions might undermine the most crucial sustainability requirements and provide solutions that corporations determine to the degree they can invest in such initiatives (Partzsch et al., 2019). While we could not identify a specific antagonist regime coalition that rejects the sustainable development agenda or actively delegitimises it, it is evident that regime players would *provide a framing that would not clash with the market logic*. For instance, several fast-fashion brands have recently been scrutinised in the UK due to their business model. These companies actively defended their business model, which is based on cheapness of items and the fastness of delivery, often at the cost of employee well-being and fair supplier cooperation (Eley, 2018). This is also evident in the recent fallout between the Norwegian Consumer Authority and H&M. The Norwegian authorities blamed the global brand for greenwashing. They found H&M’s Conscious collection marketing misleading statements on sustainability credentials (Hitti, 2019). Bente Øverli, deputy director-general at Norway’s Consumer Authority, argued that “H&M [is] not being clear or specific enough in explaining how the clothes in the Conscious collection and their Conscious shop are more sustainable than other products they sell…Since H&M [is] not giving the consumer precise information about why these clothes are labelled Conscious, we conclude that consumers are being given the impression that these products are more sustainable than they actually are” (Dwyer, 2019).

Most regime players would engage with sustainability through the lens of 'business case' in a more instrumental manner (Gao & Bansal, 2012; Hahn et al., 2015; Joseph et al., 2019). Thus, it is important to highlight that we do not propose pure sustainability or market logic, but rather highlight that the field is complex, and regime players are exposed to both logics simultaneously. While slow and sustainable fashion developments have presented various alternatives at the niche level, “the challenge now is extending the slow concept on a larger scale”; hence the regime's transition to sustainable fashion (Clark, 2015, p. 444).

Based on the above, we argue the following:

*Interaction 1b—Regime players provide their framings to shift or reinforce the dominant logic in the landscape using the space of protagonist or antagonist coalitions. Protagonist coalitions may provide eco-efficiency framings to shift the dominant market logic in the landscape that guides the regime practices. However, this may entail greenwashing if and when the corporate action and language are decoupled. Antagonist coalitions may provide fastness and ultra-fastness framing further to reinforce the legitimacy of market logic in the landscape. Negative impact niches may also provide eco-efficiency framings to legitimise their practices and operations.*

### Landscape-niche interactions

Like protagonist and antagonist regime coalitions, it is vital to highlight the distinctions between *negative and positive impact niches,* as we explained earlier. Like the regime players, niche players operate in an institutionally complex environment where both environmental and social sustainability logic, as well as the market logic, guide beliefs, norms and practices. However, negative and positive impact niches are predominantly guided by one logic more than the other (i.e. demonstrating one dominant logic rather than being impacted by both logics equally).

#### Landscape–Niche Interactions (2a)

*Market logic dominates the negative impact niches*. An example of a negative impact niche in the fashion industry is ultra-fast fashion that “can bring products from design to sale in as little as a few days, focusing on rapidly responding to consumers' increasing demands for immediacy and fashionable innovation” (Camargo et al., 2020, p. 538). Ultra-fast fashion players like Boohoo and Shein use digital technologies to gather and utilise big data on consumer behaviour, personalise shoppers' experiences, and heavily draw on social media and influencers (Monroe, 2021). Even during the Covid-19 crisis, these companies were reportedly “quick to capitalise on Covid-19 as an opportunity to boost sales, but had paid little attention to low wages and unsafe working conditions in its suppliers' factories […and] growth and profit were prioritised to the extent that the company lost sight of other issues” (Monroe, 2021, p. 2). A recent article in the Guardian demonstrates how these ultra-fast fashion players, such as Shein, exacerbate the problem of unsustainable consumerism while being responsible for environmental and social sustainability problems. For instance, “some workers at factories supplying Shein reported working more than 75 h a week. In one of them, workers got one day off a month” (Mahmood, 2022). These niche players maintain legitimacy as their operations and business models align with market logic which has dominated the fast fashion regime in decades.

Some negative impact niches may not appear negative at first sight. However, they still operate in alignment with the market logic. An example is platforms such as Depop, which may be regarded as “important services that can drive the [circular economy] for fashion” by some scholars (Manieson et al., 2021, p. 342). However, the same platform can also be critiqued for its social implications, as it still encourages unsustainable consumption, leads to fashion addiction and exacerbates social inequality and gender issues (Hitchings-Hales, 2021; Lieber, 2019; Mahmood, 2022). Here, too, we observe that while some niche players may posit themselves as more sustainable compared to the regime players, these niche players also carry attributes of the dominant market logic in various ways and, unfortunately, reinforce unsustainable and unethical consumption patterns, exacerbating both environmental and social challenges.

On the other hand, s*ustainability logic dominates the positive impact niches.* However, sustainable fashion players within the positive impact niches must satisfy the economic bottom line to ensure funding and maintain operations whilst creating a positive impact aligned with their mission (Illge & Preuss, 2012). This is why clashes between different logics are often observed in sustainable business models (Bidmon & Knab, 2018; Smink et al., 2015a, 2015b). This is also evident in the work of sustainable fashion entrepreneurs, who are often challenged by the conflicting demands of maintaining a financially successful business while at the same time aligning this business with their ethical and sustainable values (Heinze, 2020). Indeed, a recent study portrays how sustainable fashion entrepreneurs experience financial precarity and tension “between pro-social motives and more traditional, masculine or “gladiatorial” entrepreneurial tendencies […] that privilege competitiveness and financial reward, offering another component of emotional complexity experienced by [sustainable fashion] entrepreneurs”. Therefore, the growing literature on social/environmental enterprises explores mission drifts—“the risk of losing sight of their social missions in their efforts to generate revenue” (Ebrahim et al., 2014, p. 82). Mission drifts demonstrate that positive impact niche players face the risk of drifting apart from their missions if market logic gains dominance.

Based on the above, we argue the following:

*Interaction 2a—Landscape diffuses market and sustainability logics to the regime players, providing institutional complexity at the niche level. While market logic dominates negative impact niches, i.e. ultra-fast fashion, sustainability logic dominates the positive impact niches. However, positive impact niches face the risk of mission drifts due to the tensions embedded in their business model that reflect the contradictions between market and sustainability logics.*

#### Niche–landscape interactions (2b)

In response to the environmental and social challenges brought by the fast fashion regime, positive impact niche players emerged in recent years to challenge the dominant practices and norms with novel business models (Lueg et al., 2015; Stål & Corvellec, 2018). Earlier, we introduced six framings of these positive impact niches that challenge the fast-fashion regime. We observe many players within the positive niches that engage in these framings, demonstrating *alignment with the sustainability logic, albeit diagnosing different aspects of fast fashion as problematic or proposing alternative solutions to the challenges faced by the fashion industry.* Drawing on *consistency framing*, California Cloth Foundry legitimises itself by suggesting that “our skin absorbs what we put on it” and hence, “[they] choose only botanical ingredients and avoid all petrol-based fibres, treatments and dyes” (California Cloth Foundry, 2021). Utilising the *degrowth or sufficiency framing*, Petit Pli designs children's clothing items considering the growth rate of children using new materials that enable continuous size adjustments, aiming to reduce the consumption of children's clothing through degrowth principles. Abrazo Style—a company that promotes *ethical framing*, describes its products as “fairly traded, quality, hand embroidered, 100% cotton Mexican apparel in contemporary fabrics and designs” (Style, 2021). Similarly, other sustainability entrepreneurs use *ethical framing* to highlight the social dimension of sustainability and emphasise providing jobs for communities and local economies guided by social inequalities in their businesses (Su et al., 2022). Advocating *slow framing*, the Tiny Closet creates clothing from outsourced/deadstock fabric while ensuring all pieces are made to order, thereby aligning itself with the principles of slow fashion (Wardrobe Oxygen, 2021). Using deadstock fabric also aligns itself with the other framing: circular. Promoting *circular framing*, Pure Waste produces clothing items from waste fabric (Pure Waste, 2021), and Nuw provides a social network to share clothes with people in your local community and extend the life cycle of our wardrobes (Nuw, 2021).

Unlike the positive impact niches, however, the negative impact niches, like ultra-fast fashion, may further *reinforce the legitimacy of market logic with an unsustainable framing of ‘ultra-fastness’ demonstrating an alignment with the market logic*. They engage in discursive battles to hook customers to buy more with aggressive online engagement and an abundance of style while at the same time staying efficient with a small inventory and no brick-and-mortar stores (Camargo et al., 2020; Monroe, 2021). In an interview, Carol Kane and Mahmud Kamani, the co-founders of Boohoo, explain the foundations of their business by suggesting they saw the internet as a way “to cut out the middleman and market directly to customers” and in 2006, they set up Boohoo. The partnership [between Kane and Kamani] covered all the bases: [Kamani] had the money, while Ms Kane knew the industry and the tastes of fashionable young women” (Lewis, 2020). Thus, the foundations of these businesses already reflect the dominant market logics. However, while sustainability logic may not dominate ultra-fast fashion players, they may also engage in eco-efficiency framing from time to time. Since they, too, are exposed to sustainability logics. For instance, Boohoo announced a collaboration with media personality Kourtney Kardashian Barker and launched new collections focusing on sustainability. However, “many accused Boohoo and Kourtney of greenwashing—which is when a company positions itself as being environmentally friendly in marketing but lacks eco-conscious practices” (Wheeler, 2022).

Based on the above, we argue the following:

*Interaction 2b—Niche players provide their framings to shift or reinforce the dominant logic in the landscape. Positive impact niches provide their pro-sustainability framings (consistency, degrowth-sufficiency, ethical, slow, circular and sharing) to shift the dominant market logic in the landscape that guides the regime practices. Negative impact niches provide framings, like ultra-fastness, to further reinforce the legitimacy of market logic in the landscape. Negative impact niches may also provide eco-efficiency framings to legitimise their practices and operations. However, this may entail greenwashing if and when the corporate action and language are decoupled.*

### Regime-Niche Interactions (3a&b)

Based on examples from fashion, the nature of relationships between the regime and niche players can be characterised as *coopetitive* because we observe that regime and niche players engage both in cooperative and competitive framing. Regimes and niches may copy the framings of the niches or position themselves as competitors to some niches, which we refer to as competitive framing. Alternatively, both regime and niche players may collaborate with each other and position themselves as partners (Dzhengiz et al. 2023), which we refer to as cooperative framing.

The tragic Rana Plaza event and the regulatory environment, such as the EU circular economy regulations, pushed the fast fashion incumbents to partially adjust to the niche instead of heavily investing in the current paradigm. H&M joined forces with the Ellen MacArthur Foundation and committed to a 100% circular vision with the “goal to only use recycled or other sustainably sourced materials by 2030” (H&M, 2017). They added that “[they] are aware that [this] vision means a big change from on how fashion is made and enjoyed today and if [they] want to take the lead in this challenge” (H&M, 2017). A similar player is VF Corporation, which introduced takeback systems to reuse and recycle their products (VF Corp., 2017). These show that fast fashion incumbents are copying the pro-sustainability framings of niche players and getting into direct competition with many positive impact niche players, thus engaging in competitive framing. Because, due to these initiatives, they can communicate with the consumer where they are as the industry’s incumbents regarding sustainability and target a portion of the market for environmentally conscious consumers.

Positive impact niches may also use competitive framing to legitimise themselves and their sustainable technologies or business models (Kishna et al., 2017). They use various discursive strategies to delegitimise the regime players framing the regime “as insufficient, outdated, irresponsible, or unacceptable” (Geels, 2010, p. 506). For instance, the founder of Nuw highlights that “[she] was angry and frustrated that [she] had been so complicit in an industry that caused so much harm, and [she] was heartbroken because [she] did not feel [she] could enjoy fashion without contributing to the problem” (Nuw, 2021). Similarly, Batshava Hay, an entrepreneur that focuses on recycling existing fabrics to produce new clothes, reminds us that “[their] main issue is with companies like H&M pushing such huge quantities of cheap clothing by calling it sustainable, yet in the end, they are still producing a massive collective waste…the focus should really be on buying less and wearing what you own over and over again, rather than buying too much cheap, disposable clothing” (Dixon, 2019). Here, we observed the delegitimisation efforts of positive impact niches as a response to the uptake of sustainability logic by the regime players, using a competitive framing.

On the other hand, competition with the fast fashion regime is fierce for negative impact niches. Boohoo, the ultra-fast player, recently bought brands such as Debenhams and Dorothy Perkins. Its executive chairman, Mahmud Kamani, said ("Debenhams shops to close permanently after Boohoo deal," 2021): "This is a transformational deal for the group, which allows us to capture the fantastic opportunity as e-commerce continues to grow. Our ambition is to create the UK's largest marketplace.[…] Our acquisition of the Debenhams brand is strategically significant as it represents a huge step which accelerates our ambition to be a leader, not just in fashion e-commerce, but in new categories including beauty, sport and homeware." As the statement shows, strategies such as mergers and acquisitions and demonstrating interest in market leadership through competitive framing show the efforts of negative impact niches acting upon the dominant market logic to shape the regime further using a competitive framing.

The relationship between niche and regime players is complex due to their horizontal and diagonal linkages (Ingram, 2015). The dynamics, therefore, are not always competitive. Sometimes, “incumbent players lose faith in the regime due to much landscape pressure and no longer defend the regime” (Smink et al., 2015a, 2015b, p. 88). Such appears to be the case in fast fashion. We identified several examples of cooperative interactions: strategic alliances between the regime and niche players and innovation ecosystems whereby incumbents develop, fund, and cooperate with startups. For instance, Lenzing and H&M joined forces for the Conscious Collections in 2011. H&M uses Lenzing's Tencel branded Lyocell fibres- based on a niche technology that produces fibres from natural wood pulp from sustainable tree farms and biomass (H&M, 2017). H&M also partnered with I: Co, which collects clothing and footwear for reuse and recycling (Reuters, 2019). Bext360, a blockchain startup, partnered with various regime incumbents such as C&A, Zalando, PVH Corp and the Kering Group to pilot their blockchain technology to track and trace the value chain of organic cotton (Knapp, 2019). Kering—the global fashion conglomerate with a portfolio of Gucci, Saint Laurent, and Balenciaga brands—is working with the startup accelerator Plug and Play to launch the Kering Sustainable Innovation Award. This award aims “to boost Chinese startups with the potential to bring a positive environmental and social impact to the apparel and textile industries” (van Elven, 2018). In summary, incumbents employ competitive strategies by adjusting slowly and copying the strategies of positive impact niche players that demonstrate novel pro-sustainable framings. Simultaneously, they collaborate with and enable the development of positive impact niche players, which is a helpful strategy to remain competitive, as the legitimacy of positive niche players enhances over time.

Based on the above, we argue the following:

Interaction 3a*- Regime players may copy the pro-sustainability framings (i.e. circular framing) of positive impact niches by engaging in competitive framing, reinforcing the legitimacy of sustainability logic. Regime players may also engage with cooperative framing and actively collaborate with positive impact niches, reinforcing the legitimacy of sustainability logic.

Interaction 3b*- Positive impact niche players may use competitive framing to infiltrate the regime and gain dominance by delegitimising dominant regime players through an emphasis on how the market logic still dominates the regime players, which is evident in various examples of greenwashing of regime players. Alternatively, some positive impact niche players may also engage with cooperative framing and even actively collaborate with regime players to infiltrate the regime and gain dominance. Negative impact niche players may use competitive framing and engage in strategic moves, such as mergers and acquisitions aligned with the market logic, to further shape regime practices, infiltrate the regime, and gain dominance.

### Regime interactions (4)

Examples from fast fashion demonstrate that the regime is filled with persistent institutional tensions and contradictions amongst the players, which is reflected in various controversies and framing contests. For instance, in 2021, the International Sericultural Commission (ISC), an inter-governmental organization that aims to develop the silk industry worldwide, registered an official complaint to the Federal Trade Commission (FTC) about silk's score on the Higg Index of the Sustainable Apparel Coalition (Mathews, 2020). Higg Index is a standardised measurement of value chain sustainability (Sustainable Apparel Coalition, 2021). Sustainable Apparel Coalition helps fast-fashion players legitimise their eco-efficiency framing by developing the Higg Index to measure and reduce sustainability impacts in the textile value chain (Sustainable Apparel Coalition, 2021). The complaint claimed that silk as a fibre is unfairly and inaccurately represented on the Higg Material Sustainability Index (MSI). This shows that there are framing contests in the fast fashion regime due to controversies over the sustainability of different materials.

Similar framing contests have also been observed regarding plastic-based fibres' sustainability and environmental impacts, e.g. polyester. The Fossil Fashion report, produced by the Changing Markets Foundation, showed that more than half of all textiles produced globally contain polyester, which”bare [many] challenges of recycling once it has been blended with other materials”; thus,”the contribution of fast fashion to waste and of the microplastic pollution which synthetic clothing sheds when washed” (Edie Newsroom, 2021). This report challenged fast fashion incumbents of the regime, such as Nike, H&M, Primark and Zara, by revealing that” clothes made from recycled plastic bottles are just as damaging to the environment” ().

Thus, independent nonprofits and non-governmental organizations (NGOs) played an essential role in these regime dynamics (Fischer & Newig, 2016). Another example is the Clean Clothes Campaign which “repeatedly addressed moral and ethical aspects in fashion production [..] and put pressure on fashion companies to minimize negative social and environmental impacts” (Beyer & Arnold, 2022, p. 46). Here, the emotional framing efforts of NGOs (Dzhengiz et al., 2021) can be effective in further pushing the dominant companies in the regime to further enhance their social and environmental sustainability standards, for instance. Another campaign as such is the Detox campaign of Greenpeace. For instance, Adidas has been scrutinised by Greenpeace “even after the company’s commitment to [Greenpeace] detox campaign and only after setting a credible roadmap, has [Greenpeace] announced these commitments as a victory for the industry” (Dzhengiz et al., 2021, p. 2477). Here, NGOs such as Greenpeace are not dominant regime players (like the incumbents of fast fashion). However, they act as challengers to shift the regime dynamics by interacting with other regime players through campaigns.

Alternatively, regime players may act aligned, as mentioned earlier in the regime coalitions. Industry-policy cooperations, voluntary agreements, and commitments are also common in fashion. For example, Textiles 2030 is a voluntary agreement for the fashion industry to transform industry practices by reducing companies’ impacts on climate change, whereby many dominant regime players such as Marks and Spencers, Primark, and Next are members (WRAP, 2021). Another example focuses on a social sustainability issue, labour conditions. The Better Work Programme, organised by International Labour Organization and International Finance Corporation, invites textile factories to improve working conditions with a vision to lift people out of poverty in the global garment industry by providing decent work, empowering women and promoting inclusive economic growth (Better Work, 2022). We provided several examples of these kinds of protagonist coalitions in fast fashion earlier to explain how through these coalitions, they provide their framing to shift or influence the dominant logics in the landscape. Here, we further add that such coalitions provide a space to negotiate over the different pro-sustainability framings of different regime players, thus also a platform for regime interactions.

Based on the above, we argue the following:

*Interaction 4- Regime players may propose misaligned framings, thus leading to framing contests. Alternatively, they may propose aligned framings, leading to coalitions, whether protagonist or antagonist.*

### Niche Interactions (5)

In the context of fast fashion, we introduced positive impact niches that engage with pro-sustainability framings (consistency, degrowth-sufficiency, ethical, slow, circular and sharing) and negative impact niches that engage with unsustainable framings (i.e. ultra-fast fashion). Both negative and positive impact niches interact amongst and with each other to some degree.

Recently, ultra-fast fashion started infiltrating the dominant regime, even threatening the dominant regime players such as H&M and Zara with their ultra-fast offerings (Monroe, 2021). As a niche, ultra-fast fashion threatens the viability of rising pro-sustainable framings of positive niche players. The overconsumption embedded in the business models of ultra-fast-fashion players like Shein or Boohoo is viewed as fundamentally unsustainable and unethical (Nguyen, 2021). Positive impact niches, through their pro-sustainable framings that aim to address the environmental and social sustainability challenges, compete with the negative impact niches' unsustainable framing. For instance, some critics argue that the ultra-fast negative impact niches promote the linear economy, often discard the ethical dimension of designing and marketing products, neglect paying fair wages to workers, and are associated with poor working conditions in their supply chains (Kent, 2020). This controversy between the existing niches is seen in social media, where “sustainable fashion influencers are competing with a major force capturing the attention of Generation Z and millennial women: the ultra-fast fashion industry” (Birenbaum, 2021). Also, many entrepreneurs, like Tom Cridland, are fighting against ultra-fast fashion, offering durable products, and challenging the notion of ultra-fastness with quality, durability, and sustainability (Ford, 2019). Cridland offers the ‘30 Year Collection’ as an antidote to fast and ultra-fast fashion and encourages customers to “hold on to [their] clothing for a lifetime” (Ford, 2019). Another sustainability entrepreneur, Anne-Marie Tomchak, critiques the Instagram and Tiktok marketing heavily adopted by ultra-fast fashion players such as Boohoo and Shein and says that “I am personally of the view that the advertising model around social media is contributing to climate change because it has led to the acceleration of fast fashion” (). Here, we observe how positive impact niche players may contradict and challenge negative impact niche players through framing the unsustainability of the business models that rely on ultra-fastness.

Between different positive impact niche operators, cooperative framings are also in play. A British blogger Francesca Willow reported about cooperation around the sustainable clothing bloggers and brands (Willow, 2017): “We do not believe in competition in our community (we like each other too much for a start), so we decided to join forces, bringing together our resources and ideas—enabling us to talk about brands we believe are making a difference, whilst also getting the chance to work together.”

In the context of new sustainable material innovations, Finland has been reported to build up an entrepreneurial ecosystem around cellulosic textiles. “Collaboration across the value chain has been essential for accelerating innovations,” says Dr. Solveig Roschier from Gaia Consulting (Business Finland, 2018). This is not to say that positive impact niches would not also have competing framings. As the definitions in Table 1 showed earlier, the proponents of different pro-sustainable framings may prioritise different issues. For instance, while ethical framing prioritises the social dimension of sustainability, circular framing prioritises resource use and circulation. We were able to identify examples between positive and negative impact niches and cooperative framings amongst positive impact niche players and competitive framings amongst positive impact niche players. However, we could not find examples of cooperative and competitive framings between different negative impact niche players. While these relationships may exist in real life, we are limited in searching for cases representing these relationships.

Based on the above, we argue the following:

*Interaction 5- Positive impact niche players may use competitive framing and engage in framing contests to infiltrate the regime and gain dominance by delegitimising negative impact niche players through an emphasis on the unsustainability of ultra-fast fashion business models relying on market logic. Alternatively, some positive impact niche players may also engage with cooperative framing and even actively collaborate with each other to infiltrate the regime and gain dominance.*

## Discussion

Based on the illustrative case of (un)sustainable transitions in fashion, we built a conceptual framework, as shown in Fig. 1 and proposed several arguments in the previous section supporting this framework. In this section, we further explain how our framework resonated with the extant MLP literature and in what ways the illustrative case of fashion transitions helped us further expand the existing conversation on MLP and sustainability transitions. We do so by discussing each interaction we detailed with various examples in the previous section. We conceptualised five distinct interactions between landscape and regime players, landscape and niche players, regime and niche players, amongst different regime players and niches.

Figure 1 demonstrates two interactions between the landscape and the regime players (1a and 1b). One (1a) focuses on the diffusion of complex and heterogenous institutional logics, both market and environmental and social sustainability logics, to dominant regime players. This interaction aligns with the existing transition literature that focuses on the MLP. For instance, Geels and Kemp (2007, p. 446) highlight that “landscape developments create pressure on the regime, leading to major problems”. They argue that while regime players try to react to landscape developments with incremental changes, they often cannot fully solve the problems; hence, niche players find a window of opportunity (Geels & Kemp, 2007). They further argue that landscape-regime interactions provide room for socio-technical transitions; however, they also highlight that the landscape changes over a long time.

The other interaction between landscape and regime (1b) emphasises the agency of regime players to influence the landscape by legitimising different logics through their own framing efforts, often through developing protagonist or antagonist regime coalitions. Scholars highlight that “institutions also depend on agency; [since] institutions are constructed by the very same players” (Smink et al., 2015a, 2015b, p. 89). This agency is thanks to regime players' framing efforts which give sense to other stakeholders and push their meaning systems at the landscape level. A recent article by Geels (2020, p. 9) captures this interaction as “structural elaboration”, which he defines as ‘upward’ actions that reproduce existing institutions ("morphostasis") or transform them ("morphogenesis")”. He notes that such agency can often be attributed to institutional entrepreneurship of professional societies, industry associations, and standardization organizations. In the case of fashion, such organizations include but are not limited to Fashion for Good, Partnership for Sustainable Textiles, and Sustainable Apparel Coalition. Still, we find that while Geels (2020) recently captured this agency of such organizations to influence the landscape, MLP literature overall did not focus much on how these organizations would be able to do so by using various framing strategies. Here, we believe our paper can start a new conversation by inviting other scholars to explore the role of agency through protagonist and antagonist coalitions.

Figure 1 demonstrates two interactions between the landscape and the niche players (2a and 2b). Like in the landscape-regime interactions, we also include both the top-down and the bottom-up dynamics. One interaction focuses on the diffusion of complex and heterogenous institutional logics, both market and environmental and social sustainability logics, to positive and negative niche players in a top-down fashion. Albeit differently because we explain positive and negative niches are dominated by one logic more than the other (sustainability logic for positive niche, market logic for negative niche). Thus, the landscape also shapes the niche level through market and sustainability logic diffusion. Therefore, like the regime, niche players also operate in institutional complexity. Niche players also influence the landscape by framing to give sense to other stakeholders and push their meaning systems at the landscape level. MLP framework also acknowledges that the discursive activities of niche players (i.e. framing) “eventually result in cultural repertoires at the landscape level” (Koistinen et al., 2018, p. 113). However, as El Bilali (2019b, p. 358) highlights, with a few exceptions like Koistinen et al. (2018), MLP literature also often emphasises how the landscape provides the structure for niche players, and there is “more room for agency within the MLP.”

Figure 1 also demonstrates the regime and niche players' interactions, focusing on the competitive and cooperative framing dynamics. We explain that both positive and negative niche and regime players engage with competitive or cooperative framing to gain or maintain regime dominance. Again, these competitive and cooperative framing efforts also capture how regime and niche players actively use different framing strategies and contests to either remain legitimate and relevant (for regime incumbents) or to gain legitimacy to dominate the regime (for niche). Especially the niche players are trying to replace the regime, especially as a dominant framing emerges from framing contests between different niche alternatives (3a). On the other hand, regime players aim to maintain their legitimacy in the face of public scrutiny due to scandals and greenwashing accusations by adopting the eco-efficiency framing, which aims to combine the market logic and sustainability logic, respectively, through a business case approach (3b).

Here, our findings from fashion transitions resonate with the transitions literature which gave significant attention to regime-niche interactions. This literature posited that as the niche network expands, the new offering of the niches gains significant market share (Geels & Schot, 2007). Therefore, regime-niche interactions are often viewed as competitive. Regimes may resist niche players by using their power to access resources (Koistinen et al., 2018). This resistance is often viewed when they heavily invest in the current paradigm or partially adjust to niches (Smink et al., 2015a, 2015b). For instance, the regime may use the financial system to control which niche innovations would receive future investments, highlighting that only those niche players that fit or conform with the regime would gain sufficient legitimacy to replace the regime (Geddes & Schmidt, 2020). Regime players may respond to niches through de-alignment strategies that are competitive and defensive and re-alignment strategies that are cooperative and proactive (Smink et al., 2015a, 2015b). Thus, our findings add to the literature on MLP in transitions by showing that regime-niche dynamics are indeed coopetitive.

Figure 1 also demonstrates interactions within the regime by demonstrating how regime players may have framings that align with each other, which may result in regime coalitions, whether protagonist or antagonist. They may also posit misaligned framings, which may compete with each other, even clash, resulting in framing contests. Literature on transitions also portrayed regimes as semi-coherent entities that entail endogenous sources of change due to multiple institutional logics that offer different behavioural rationalities (Runhaar et al., 2020). Thus, our proposal of regimes as a framing contest field demonstrates alignment with the extant literature. Joining Fuenfschilling and Truffer (2014), we also argue that regimes are filled with persistent institutional tensions and contradictions amongst the players. In the fast fashion regime, we demonstrated these contradictions, especially in the recent controversies about different materials, including silk and polyester which destabilised the regime and many dominant regime players are still unable to clearly defend their position (for instance, for marketing recycled polyester as sustainable and green).

Finally, Fig. 1 also demonstrates interactions within the niche players by demonstrating how positive impact niche players may use competitive or cooperative framing amongst themselves to legitimise each other and engage in framing contests with negative impact niche players to infiltrate the regime and gain dominance by delegitimising their model of business. This, too, was highlighted by the MLP literature, which emphasised how niche players may compete to gain dominance and have their novelties used in the regime or even replace regime practices and norms (Schot & Geels, 2008). For instance, like our emphasis on coopetitive dynamics between different positive impact niche players, Köhler et al. (2019) also highlighted the need to understand the complementary and competing interactions between emerging and existing niches. Here, however, we expanded the conversation within the MLP literature on transitions at the niche level. Exclusively, we propose a distinction between negative and positive impact niches and challenge the assumption that niches are almost always associated with positive environmental and societal impact.

Unlike the expectation of a ‘sustainable transition’, we show that fashion first evolved from a prêt-à-porter to fast fashion. Today, the fast-fashion regime is threatened mainly by the negative impact of ultra-fast fashion niches, unlike the expectation of sustainable fashion players taking over the regime. Indeed, with all the stakeholder concerns, ultra-fast players like Boohoo have been financially very successful and still growing via takeovers and acquisitions of many known fast fashion brands.

## Conclusions

In this paper, we shed light on (un)sustainable transitions by presenting an illustrative case of transitions in fashion. Based on the illustrative case of fashion, we presented a framework that integrates institutional logics and framing with the interactions in the MLP. We also explored the fashion industry's positive and negative sustainability developments. By doing so, we contributed to the literature in three distinct ways.

First, we demonstrated how fast fashion became the regime and how ultra-fast fashion will likely replace today’s fast fashion. Antal et al. (2020) previously invited others to study unsustainable trends, which have received scant attention thus far. Our analysis particularly focused on these unsustainable trends as we highlighted the negative impact niches at the micro level, antagonistic coalitions at the meso level, and the framing contests that include unsustainable promotion of ultra-fastness and fastness and micro and meso levels. Therefore, we answered the call of Antal et al. (2020) and contributed to the literature on MLP by expanding the conversation from sustainability transitions to unsustainable transitions.

More specifically, concerning our first contribution, we challenged the notion that niche developments often lead to sustainable solutions. We joined scholars such as Næss and Vogel (2012), who referred to 'unsustainable niches' and defined two categories of niche players based on their sustainability impact: positive impact and negative impact niche players. While many other scholars gave examples of positive impact niche players in fashion and other contexts, we demonstrated ultra-fast fashion as a negative impact niche. Doing so, we also contributed to the literature on MLP by extending the notion of niche.

Secondly, we shed light on the transitions of the previously neglected fashion context. To our knowledge, transition studies have failed to study the evolution of the textile, apparel, clothing and fashion industries thus far. Therefore, we believe our analysis, based on secondary data and illustrative cases, can provide an initial understanding of transitions to fast fashion (and currently to ultra-fast fashion).

Thirdly, to explain interactions in the MLP framework, we draw on framing contests and institutional logics literature. We, therefore, joined other studies that created a bridge between transitions and framing and institutional logics (Fuenfschilling & Truffer, 2014; Runhaar et al., 2020) and elaborated on the idea of agency and power, which extant MLP literature in transitions was critiqued for lacking (El Bilali, 2019a, 2019b; Svensson & Nikoleris, 2018).

### Limitations and future research guidance

Our study is not without limitations. Firstly, due to our limited research focus, we did not provide how consumer demands may shape the (un)sustainable fashion transitions. Based on Fischer and Newig (2016), changing consumer trends towards more sustainable alternatives is likely one of the explanatory factors for landscape change today. Here, we believe future research should further focus on the role of consumer trends in shaping the landscape, thus, the macro level of fashion transitions and explain to what degree the changes in consumer trends affected the institutional logics that provide legitimate norms, practices and behaviour.

Secondly, our research is based on secondary data and illustrative examples. While these examples helped us identify some adverse developments and unsustainable transitions, we believe future empirical research is necessary to shed light on the negative impact niche players further, mainly to show how they use competitive framing amongst themselves and engage in framing contests with regime players. Exploring the framing strategies of ultra-fast fashion players like Boohoo and Shein can be a good start for future research. Furthermore, a similar analysis of antagonistic coalitions is also necessary. Here, empirical studies could especially explore how in the face of controversies such as the one about polyester and silk in the Higg Index, different regime players may act together in defence of their positions.

To conclude, for a sustainability transition to occur, positive developments at all levels need to occur in the fashion industry. The multi-layered interaction process presented in this article shows how complex and multi-faceted the transition is in fashion. Joining (Buchel et al., 2018, p. 39), we also highlight that “to effectively contribute to a transition, one needs to acknowledge the systemic complexity, the myriad of interrelated players and scale levels, and the fact that too often the solutions we are working on now are part of the problem”. Moving forward, we invite future scholars to explore the transitions in the fashion industry, which, in our view, would provide novel explanations regarding unsustainable transitions.

### Implications for practice

Our research framework informs three types of practitioners: sustainable fashion entrepreneurs, sustainability managers in fast fashion incumbents, and policymakers. Here, we summarise the implications of our research for these practitioners.

Firstly, our framework shows that sustainable fashion entrepreneurs (that form the positive impact niche) will need to compete with each others’ framings because different entrepreneurs focus on different challenges within the unsustainability of fast fashion and propose different solutions. We demonstrated that these entrepreneurs often go beyond eco-efficiency framing and propose six distinct framings: consistency, degrowth-sufficiency, ethical, slow, circular and sharing. Ultra-fast fashion and fashion players also increasingly adopt eco-efficiency, circular, and sharing framing. Therefore, sustainable fashion entrepreneurs will need to actively challenge fast and ultra fast fashion to establish their legitimacy and destabilise the unsustainable transitions. The idea of framing contests provides sustainable fashion entrepreneurs with an idea of how they can use language tactics, emotions, and ethical values, to replace dominant regime practices and negative impact niches.

Moreover, the tensions between sustainability's environmental, social and economic demands challenge sustainable fashion entrepreneurs. We join Heinze (2020) and note that financial precarity may affect these entrepreneurs if they cannot balance the demands of the market and sustainability logics. Here, sustainable fashion entrepreneurs must be cautious of drifting away from their missions if market logic gains dominance in their operations.

Secondly, our framework provides insights for sustainability managers in fast fashion incumbents (as part of the regime). We demonstrated that fast fashion incumbents had experienced a legitimacy crisis since the Rana Plaza tragedy. Here, a way forward for the incumbents has been cross-sector partnerships and inter-firm alliances to address various sustainable development goals (Dzhengiz 2020) often collaboratively with others in the industry through protagonist coalitio (Dzhengiz et al. 2023). We underline that sustainability managers in fashion incumbents play a crucial role as internal change agents and external advocates of their company’s, framing their own sustainable solutions, which are often associated with eco-efficiency. As internal change agents, we believe these managers should motivate their organizations to integrate sustainability into the core of the business. This will, fundamentally, require challenging the fast fashion business model and its emphasis on over-consumption. Here, framing and framing contests, not for external legitimacy but for internal organizational change, can help these managers (Girschik, 2018).

Finally, our study posited how transitions could turn unsustainable and not all niches require protection for innovation's sake, especially when their impact on the natural environment and societal well-being is negative. Thus, joining Lazarevic and Valve (2020), we believe policymakers should address normative questions. Who decides what niches should be protected and on what grounds? Should they be protected at the expense of others? Policymakers should think about these questions. Our framework can help policymakers to conceptualise different multilevel interactions in the context of fast fashion and guide them on the complexities of transitions.

## Data Availability

Not applicable.

## Footnotes

1 In this article, when we refer to the fashion industry, we generally mean an overarching sectoral area that includes the whole value chain from fibre production to yarn and fabric production and, finally, clothing production and retail. While we understand that Haute Couture is still a vital element in the fashion system, we focus on fast fashion since we consider it the dominant regime.
